# Unusual case of primary spontaneous hemopneumothorax in a young man with atypical tension pneumothorax: a case report

**DOI:** 10.1186/s13256-018-1732-x

**Published:** 2018-07-02

**Authors:** Youwen Chen, Zhijian Guo

**Affiliations:** Department of Thoracic Cardiovascular Surgery, Chang Gung Memorial Hospital, 123 Avenue Xiafei, Xiamen, 361028 Fujian China

**Keywords:** Primary spontaneous hemopneumothorax, Spontaneous pneumothorax, Tension pneumothorax, Thoracotomy, Thoracostomy

## Abstract

**Background:**

Spontaneous life-threatening hemopneumothorax is an atypical but treatable entity of unexpected circulatory collapse in young patients, affecting 0.5–11.6% of patients with primary spontaneous pneumothorax. Spontaneous pneumothorax is a well-documented disorder with a classic clinical presentation of acute onset chest pain and shortness of breath. This disorder might be complicated by the development of hemopneumothorax or tension pneumothorax.

**Case presentation:**

A 23-year-old Asian man was referred to the emergency room of Xiamen Chang Gung Memorial Hospital with a 1-day history of right-sided chest pain that had been aggravated for 1 hour. A physical examination revealed a young man who was awake and alert but in mild to moderate painful distress. His vital parameters were relatively stable at first. The examining physician noted slight tenderness along the right posterolateral chest wall along the eighth and tenth ribs. Primary spontaneous pneumothorax was considered, and a standing chest X-ray confirmed the diagnosis. A right thoracostomy tube was immediately placed under sterile conditions, and he was referred to the respiratory service. While in the respiratory department, approximately 420 mL of blood was drained from the thoracostomy tube over 15 minutes. Our patient developed obvious hemodynamic instability with hypovolemic shock and was subsequently admitted to the cardiothoracic surgical ward after fluid resuscitation. During the ensuing 4 hours after admission, 750 mL of blood was drained through the thoracostomy tube. A bedside chest X-ray was requested after he was temporarily hemodynamically stabilized. Primary spontaneous hemopneumothorax associated with right tension pneumothorax was considered based on the radiological impression and clinical signs. An emergency limited posterolateral thoracotomy was performed. A standing chest X-ray performed on day 6 of admission after the removal of the thoracostomy tube showed a complete re-expansion of his right lung. He remained stable and was discharged within 1 week.

**Conclusions:**

The successful treatment of a large spontaneous hemopneumothorax depends on early recognition, proactive intervention, and early consideration by a cardiothoracic surgeon. Once the diagnosis is confirmed, early thoracotomy should be considered. Such an aggressive surgery not only leads to shorter hospitalization but also confers better long-term outcomes.

## Background

Spontaneous hemopneumothorax (SHP) is a rare disorder that historically affects approximately 0.5–11.6% of patients with spontaneous pneumothorax (SP) and can be life-threatening [1, 2]. SHP was first described in 1876, and fatal cases were reported at the beginning of the last century. Non-traumatic hemopneumothorax is 30 times more common in men than in women, and the gender difference is considerably larger than that in SP [2]. Bleeding most commonly results from the rupture of a vascularized bulla or a torn adhesion between the visceral and parietal pleura [1–3]. Pneumothorax is defined as “air and frequently accompanied by a limited amount of blood or fluid in the pleural cavity” [1, 2]. SP is classified as primary or secondary. Primary SP (PSP) occurs in a previously healthy patient without underlying chronic lung disease or provoking factors, such as trauma, surgery, or diagnostic intervention, while secondary SP is associated with parenchymal lung disease, such as pulmonary fibrosis or emphysema [3]. SHP is a condition in which more than 400 mL of blood has accumulated within the pleural space in association with SP. The clinical presentation can be dramatic due to the hemodynamic instability with hypovolemic shock [4]. When blood loss is substantial, early placement of an intrapleural chest drain is necessary, and thoracotomy may be required to achieve hemostasis [1–3].

Tension pneumothorax (TP) is usually associated with mechanical ventilation or trauma, and the incidence of spontaneous development is rare. Brims reported that spontaneous TP may complicate 1.1–3.2% of unrecognized pneumothoraces and may be life-threatening [5]. TP requires emergency needle decompression and tube thoracostomy due to obvious respiratory distress and signs of cardiovascular instability.

In this report, we describe an unusual case of a young Asian man who presented to our emergency room (ER) with right-sided chest pain; he was diagnosed as having primary SHP associated with spontaneous right TP diagnosed by radiographic imaging and intraoperative findings. We discuss the clinical presentation, risk factors, and therapeutic options.

## Case presentation

A 23-year-old Asian man was referred to the ER of Xiamen Chang Gung Memorial Hospital with a 1-day history of right-sided chest pain that had been aggravated for 1 hour. He had no known medical illnesses and was well until the evening prior to presentation, at which time he developed obvious right-sided chest pain radiating to his ipsilateral shoulder with persistent chest tightness. This tightness was described as sticking in nature, significantly worse on deep inspiration and with movement, and relieved by leaning forward or lying down. There was an associated dry cough but no hemoptysis. There was no history of trauma, injury, difficulty in breathing, or palpitations. He was tall and thin and described himself as otherwise quite healthy. He had never previously been admitted to a hospital. He reported no significant chronic medical history, such as primary hypertension, any type of heart disease, disturbed microcirculation, peripheral neuropathy, diabetes mellitus, an impaired immune system, malignancies, leukemia, the long-term administration of corticosteroids, liver cirrhosis, renal failure, urinary tract infection, or hemodialysis. He also reported no history of infection, such as tuberculosis, any type of hepatitis, or acquired immunodeficiency syndrome (AIDS). There was no prior history of traumas, blood transfusions, surgical procedures, or other serious events in his medical history. He had not lived in an epidemic area and had no history of toxin or radioactive exposure. He denied a personal or family history of bleeding diathesis but reported a 10-year history of smoking 8–10 cigarettes per day. He was an office worker by occupation. He had experienced similar symptoms on one occasion 4 years previously. No abnormalities were detected at that time, and his symptoms resolved.

**Fig. 1:**
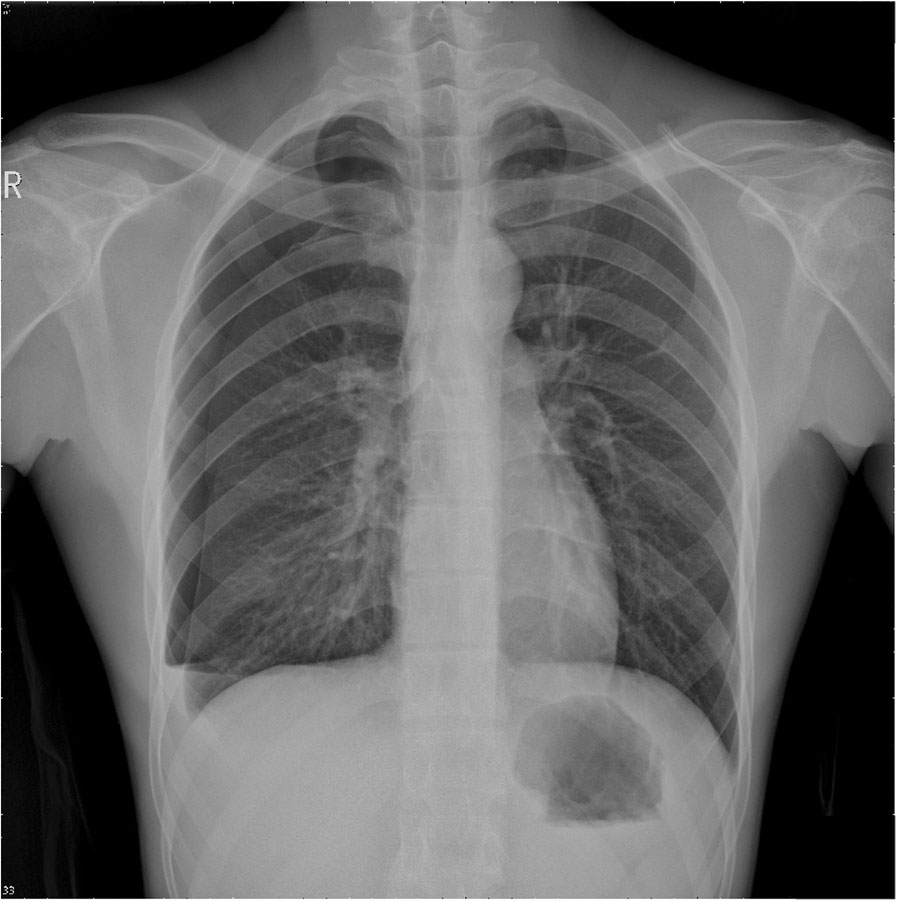
Radiographic findings of a standing chest X-ray revealed a right-sided primary spontaneous pneumothorax (approximately 30%) with a small amount of pleural effusion

**Fig. 2:**
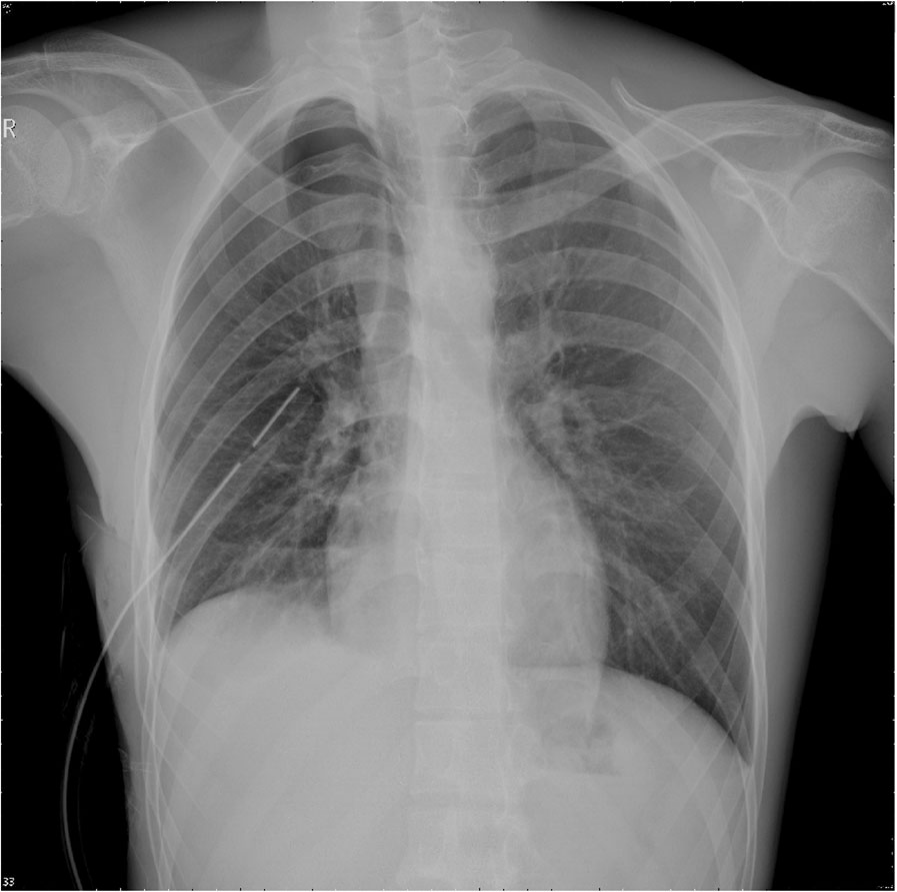
A repeat chest X-ray was performed to evaluate the position of the thoracostomy tube and the re-expansion of the right lung. A right thoracostomy tube (28-F, straight) was immediately placed under sterile conditions; approximately 50 mL light red pleural effusion flowed out from the chest tube after placement, and good fluctuation of the water column in the drainage reservoir was observed

A physical examination (PE) revealed a young man who was awake and alert but in mild to moderate painful distress. His respiratory rate was 22–26 breaths/minute with an oxygen saturation of 97%. His pulse was 96 beats/minute, his blood pressure was 115/74 mmHg, and his temperature was 36.7 °C. The examining physician noted slight tenderness along the right posterolateral chest wall along the eighth and tenth ribs. Breath sounds and percussion were documented as normal. An electrocardiogram revealed sinus rhythm with a normal axis. PSP was considered, and a standing chest X-ray (CXR) was requested. The radiographic findings revealed a right-sided PSP (approximately 30%) with a small amount of pleural effusion (Fig. 1). A right thoracostomy tube (28-F, straight) was immediately placed under sterile conditions; approximately 50 mL of light red pleural effusion flowed out from the chest tube after placement, and good fluctuation of the water column in the drainage reservoir was observed. Another CXR was performed to evaluate the position of the thoracostomy tube and re-expansion of his right lung (Fig. 2). Our patient’s vital signs stabilized, and his right-sided chest pain was apparently alleviated after chest tube placement; therefore, he was referred to the respiratory service with parenteral analgesia.

**Fig. 3:**
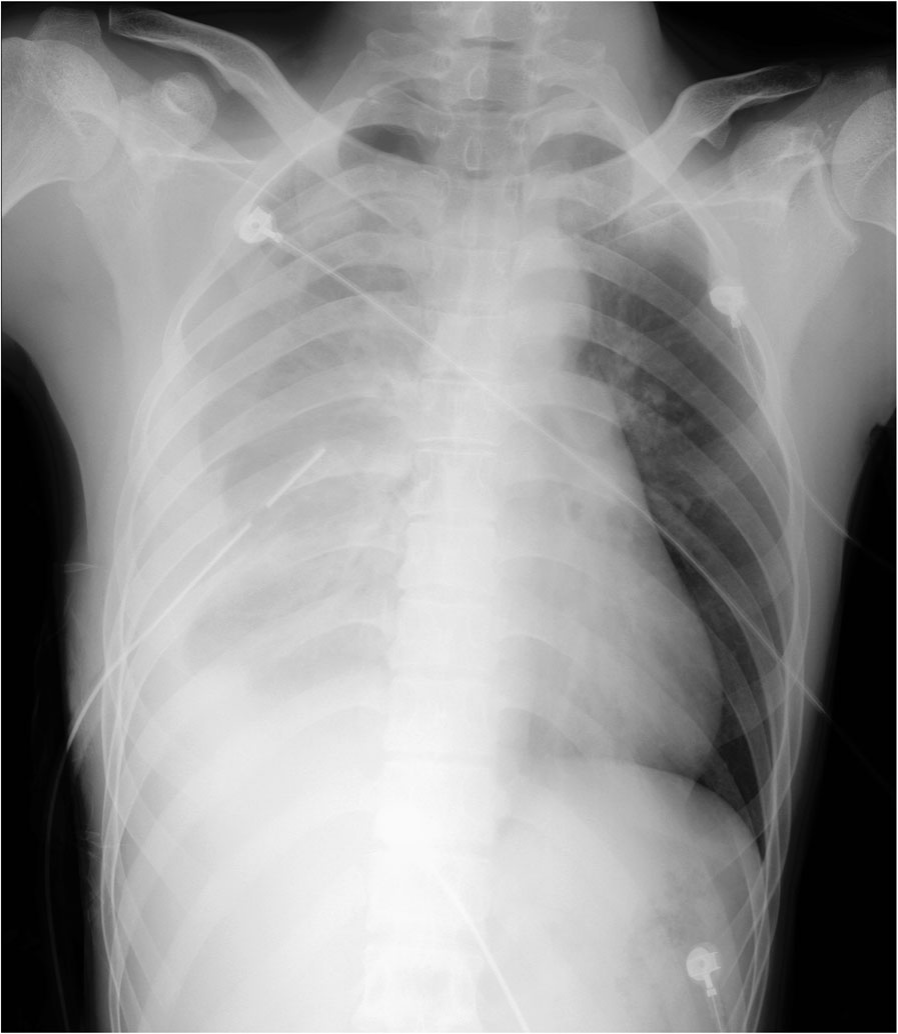
Bedside chest X-ray showing a large right-sided primary spontaneous hemopneumothorax with contralateral deviation of the mediastinum

While in the respiratory department, approximately 420 mL of blood was drained from the thoracostomy tube over 15 minutes. He developed obvious hemodynamic instability with hypovolemic shock (his blood pressure dropped from 110/70 to 75/50 mmHg), and he was subsequently admitted to the cardiothoracic surgical ward after fluid resuscitation. During the 4 hours after admission, 750 mL of blood was drained through the thoracostomy tube. His hemoglobin level dropped from 12.6 g/dL to 9.2 g/dL. A prolonged prothrombin time (PT) of 19 seconds was noted (normal reference of 14 seconds). Packed red cells and fresh frozen plasma were administered to our patient. Vitamin K (10 mg) and tranexamic acid (1 g) were also administered parenterally. He was unable to undergo an emergency chest computed tomography scan as his vital signs remained unstable after fluid resuscitation. Alternatively, a bedside supine CXR was performed when he was temporarily hemodynamic stable (Fig. 3). A primary SHP associated with right TP was considered based on the radiographic findings. A re-examination of his chest revealed markedly decreased air entry on the right side with mild tracheal deviation.

An emergency limited posterolateral thoracotomy was performed for resection of the bullae, ligation of the bleeding adhesion, and irrigation of the pleural cavity, and mechanical pleurodesis was implemented. Approximately 800 mL of blood and clots and a collapsed right lung with several apical bullae were observed. There was a small (less than 1.0 cm) tan lesion noted on the dome of the right side of his chest. Bullous apical tissues of the right lung adhering to the thoracic wall and adhesions at the region of the subclavian artery were identified as the source of bleeding. Aberrant blood vessels growing from the chest wall through the adhesion bands into the pleural lesion were thought to be torn once the lung collapsed. These bleeding blood vessels may also have arisen from the surface of ruptured bullae. Histopathology revealed that the bullous and string-like tissues were rich in blood vessels and were granulomatous.

Subsequently, the drainage from his chest tube became minimal. A standing CXR performed on day 6 of admission after removal of the thoracostomy tube showed complete re-expansion of his right lung. His hemoglobin level rose to 10.1 g/dL after a transfusion of two units of packed red blood cells, and his PT normalized. He remained stable and was discharged within 1 week (6 days postoperatively). He returned to an out-patient department for follow-up two times. The right-sided posterolateral surgical incision had healed on the 14th postoperative day. Full expansion of lungs was confirmed using a standing CXR on the 14th postoperative day and at 3 months after discharge. He was healthy as usual with no complaints or illness.

## Discussion

Although SHP is a well-documented disorder, it is rarely encountered in clinical practice. SHP is a complication of primary or secondary pneumothorax and can lead to potentially life-threatening conditions. SHP is reported to occur in 0.5–12% of all patients with SP [1, 2]. There is a male predisposition mentioned in different reports, and the reason for the relative infrequency of the incidence of SHP in females is unclear. In the review by Fry *et al.* [6], the incidence of SHP was found to be 25.4 times higher in male patients than in female patients, and they suggested that the male predisposition to hemorrhage may be due to the additional strength in exercise. The difference in incidence between men and women in SHP is much larger than that in SP. SHP occurs when greater than 400 mL of blood accumulates in the pleural cavity in association with SP. Clinically, SHP should be considered a lift-threatening emergency when a patient with a known pneumothorax develops otherwise unexplained symptoms of shock.

There are three proposed mechanisms of bleeding described in SHP cases. First, hemorrhage can result from a torn adhesion between the parietal and visceral pleurae. Second, bleeding can result from rupture of vascularized bullae and the underlying lung parenchyma. Third, bleeding can arise from torn congenital aberrant vessels branching from the pleural cupula that are distributed in and around the bulla in the apex of the lung. Only a small number of angiographic studies performed during bleeding revealed aberrant vessels of different origin [1–4, 7].

Pathological examinations reveal abnormalities around bullae or thickened bullae associated with collagenous tissue that is compatible with a torn adhesion. Examinations of the aberrant vessels show Alcian blue-positive deposits in the arterial wall and fibrosis in the intima and media. These findings are suggestive of mucoid degeneration and sclerosis, as well as an inability of the vessel to retract after disruption [8, 9]. This abnormality combined with negative intrapleural pressure leads to persistent hemorrhage into the pleural space.

A patient’s age and history, clinical PE, radiographic evaluation, air and blood aspirates, and hemoglobin levels in the aspirated blood may be very helpful diagnostic tools to differentiate between SHP and a blood-tinged effusion [9].

PSP occurs in patients with no underlying lung disease. In 81–90% of patients, subpleural bullae are found on computed tomography or at surgical exploration [5]. Factors that may be related to the occurrence of blebs, bullae and pleural porosity include malnutrition, local ischemia, connective tissue disorders, distal bronchial tree anomalies, and distal airway inflammation. An increase in pleural porosity secondary to inflammation is another proposed mechanism of the development of PSP that has been reported [2, 3, 5]. The patient in this report was a healthy, tall, thin, young man with a 10-year history of tobacco smoking. He remained at home for several hours before seeking medical care. In general, 46–50% of patients with PSP wait more than 2 days before seeing a physician, and 10% are asymptomatic. Recurrence rates range from 16 to 52% with 10 years of follow-up. Most recur within approximately 6–12 months after the first episode [2, 3, 5]. Our patient reported having similar symptoms 4 years previously, and it is quite likely that he had a previous small pneumothorax that resolved spontaneously. It is probable that previous episodes of PSP may have resulted in adhesions and put him at risk for the SHP that developed during this admission.

Primary spontaneous TP is a rare complication of PSP. Patients typically present in extremis with obvious signs of respiratory distress and cardiovascular compromise due to impaired venous return and decreased cardiac output as a result of a mediastinal shift [10, 11]. A PE can reveal decreased chest wall expansion, hyper-resonance, a displaced apex beat, tracheal deviation, decreased tactile vocal fremitus, and decreased breath sounds. Clinical signs can be absent in pneumothoraces that occupy less than 15–18% of the hemithorax. Simpson *et al.* [11] reported the cases of nine patients with PSP who were diagnosed as having TP on radiographic imaging but had no classic clinical features of TP. Holloway and Harris [12] reported four cases of PSP; in three of four patients, the diagnosis of TP was made based on a CXR. In one of the four patients, the physician was able to elicit the signs of PSP and tracheal deviation but did not believe his assessment because the patient appeared so clinically stable. In certain patients with SHP and spontaneous TP, these classical signs, such as hemodynamic instability and decreased breath sounds, can be concealed, as was evident in this patient. Therefore, a more thorough clinical PE should be performed to assess for the presence of a mediastinal shift, which may be evidenced by tracheal deviation, a displaced apex beat, and resonance over the sternum. Reversible Horner’s syndrome and distended neck veins can also be apparent in patients with TP [11]. The present case highlights the importance of obtaining a medical history and proper clinical PE and demonstrates that TP does not always present in extremis and can possibly be confused with other accompanying disorders.

Concerns and doubts are raised regarding how to assure that SHP was not caused inadvertently by the placement of the thoracostomy tube. Hemopneumothorax may be caused by the inadvertent placement of a chest tube that directly damages the rib arterial and/or venous vessels, lung tissue, or pulmonary and bronchial blood vessels. By obtaining a full clinical picture of the entire progression of our patient’s medical treatment and by participating in his resuscitation, we initially precluded the potential human factors that could have led to hemopneumothorax. More importantly, the bleeding source was demonstrated to be aberrant blood vessels rather than direct damage to the abovementioned blood vessels and/or lung tissue during the emergency limited posterolateral thoracotomy. Surgical exploration provides the most definitive and strongest evidence.

We diagnosed the patient as having right-sided SHP associated with TP based on radiographic findings, a proper clinical PE, and our observations. These observations are as follows: our patient had a first episode of PSP with a small amount of pleural effusion at the beginning of presentation. The symptoms of pneumothorax were apparently alleviated after placement of a right thoracostomy tube. He had a second episode of recurrent SP combined with atypical TP. In the progress of the secondary episode of pneumothorax, the aberrant blood vessels were torn apart because of atypical TP. Therefore, PSP led to SHP. Persistent TP caused our patient’s lung to collapse, and the collapsed lung failed to re-expand and compress the sources of bleeding, which contributed to continuous bleeding and hypovolemic shock. The placement of the proper chest tube partially relieved the thoracic pressure. However, the increase in thoracic pressure led to markedly decreased air entry into the right hemithorax with a mild leftward shift of the trachea. The abovementioned observations were evidenced in the two drainage reservoirs that were separately connected. Continuous bright red pleural effusion drained into the first drainage reservoir, and good fluctuations of water column and obvious bubbles were detected in another drainage reservoir.

Another important issue that we must address in this patient is “re-expansion” pulmonary edema, which is fluid accumulation in the tissue and air spaces of the lungs. Pulmonary edema, especially acute pulmonary edema, can lead to impaired gas exchange and may cause fatal respiratory distress or cardiac arrest due to hypoxia. Pulmonary edema is caused either by a failure of the left ventricle to remove blood adequately from the pulmonary circulation (cardiogenic pulmonary edema) or by an injury to the lung parenchyma or vasculature (non-cardiogenic pulmonary edema) [13]. The causes of “re-expansion” pulmonary edema include resolution of pneumothorax, a large amount of pleural effusion, post-large volume thoracocentesis, post-decortication, removal of endobronchial obstruction, and other abnormal conditions that effectively lead to the development of negative pressure pulmonary edema. The most common symptom of pulmonary edema is difficulty breathing, but other symptoms may occur including hemoptysis (classically seen as pink, frothy sputum), excessive sweating, anxiety, and pale skin. Shortness of breath can manifest as orthopnea and/or paroxysmal nocturnal dyspnea. Signs of pulmonary edema include end-inspiratory crackles on auscultation and the presence of a third heart sound. There is no single test to confirm that breathlessness is caused by pulmonary edema; in fact, in many cases, the cause of shortness of breath is probably multifactorial [13]. Low oxygen saturation and disturbed arterial blood gas readings support the proposed diagnosis by suggesting a pulmonary shunt. CXRs may show fluid in the alveolar walls, Kerley B lines, increased vascular shadowing in a classical batwing perihilar pattern, upper lobe diversion (increased blood flow to the superior parts of the lung), and possible pleural effusion. In contrast, patchy alveolar infiltrates are more typically associated with non-cardiogenic pulmonary edema. Lung ultrasound is also a useful tool to diagnose pulmonary edema; it is accurate and can also be used to quantify the degree of pulmonary edema, track changes over time, and differentiate between cardiogenic and non-cardiogenic edema [13, 14]. Blood tests are performed for electrolytes (sodium, potassium) and markers of renal function (creatinine, urea). Liver enzymes, inflammatory makers (usually C-reactive protein), and a complete blood count are also typically performed as well as coagulation studies: PT and activated partial thromboplastin time (aPTT). B-type natriuretic peptide (BNP) is available in many hospitals and can sometimes be performed as a point-of-care test. Low BNP levels (< 100 pg/mL) suggest that a cardiac cause is unlikely [13]. Treatment is focused on three aspects: first, improving respiratory function; second, treating the underlying cause; and third, avoiding further damage to the lung. The initial management of pulmonary edema, irrespective of the type or cause, is supporting vital functions. Therefore, if the level of consciousness is decreased, it may be necessary to proceed to tracheal intubation and mechanical ventilation to prevent airway compromise. Hypoxia may require oxygen supplementation but, if this is insufficient, mechanical ventilation may be necessary to prevent complications. Treatment of the underlying cause is the next priority; pulmonary edema secondary to infection, in most cases, requires the administration of appropriate antibiotics [14]. The patient in this report did not demonstrate the signs and symptoms of re-expansion pulmonary edema.

There are no specific guidelines for the management of SHP, and the clinical features of SHP are dramatic and depend on the volume of blood loss and the amount of air leakage [8, 9]. SHP can be life-threatening, and aggressive management is required. When SHP becomes life-threatening because of substantial bleeding, the early insertion of two large bore chest drains to evacuate the accumulated blood and air is recommended to permit the re-expansion of the lung, which results in hemostasis by compressing the bleeding vessels against the parietal pleura [7–9]. Therapeutic protocols are determined individually based on the patient’s clinical appearance and condition. The goals of treatment include resuscitation, hemostasis, and re-expansion of the lung. Initial treatment consists of resuscitation with adequate fluid replacement and drainage of the pleural space. Gradually, it has become well known and accepted that early thoracotomy has specific advantages and that the clinical outcomes in patients who undergo thoracotomy are better than those in patients managed with conservative therapy with tube thoracostomy alone. Hsu *et al.* [7] advocated tube thoracostomy alone with hemodynamically stable patients and those who have no persistent air leaks, no continuous bleeding, and no impaired lung expansion. The indications for urgent thoracotomy include an aspiration of more than 1.5 liters of blood upon the insertion of a chest drain, continued blood loss of more than 200 mL/hour, hypovolemic shock, persistent air leak, impaired lung expansion, pachypleuritis, recurrent pneumothorax, and clot empyema [7–9]. Homologous blood transfusion may also be required, although most patients do not receive this since they tend to be young and able to recover from the anemia postoperatively [7–9]. Therefore, early thoracotomy is also necessary to obtain hemostasis shortly after the onset of symptoms.

Surgical strategies for the management of PSP include open thoracotomy and pleurectomy or video-assisted thoracoscopic surgery (VATS) with pleurectomy and pleural abrasion [7–9]. An open approach offers lower recurrence rates, while a less invasive approach with VATS has the advantages of less postoperative pain, improved pulmonary function, and a decreased length of hospital stay. VATS can be performed as an elective surgery after initial resuscitation. We recognize that VATS might be the current approach of choice for the treatment of SHP. However, this patient’s family’s difficult economic conditions, lack of appropriate medical insurance, and the comparatively expensive surgical costs of VATS led us to choose a limited posterolateral thoracotomy for this patient. Currently, many real-world factors can influence the surgeon’s choice of surgical methods in mainland China. In our opinion, a limited open thoracotomy is safer in cases of hemodynamic instability in which major bleeding occurs and in “chronic” cases in which fibrin has been organized, and the gel-like pseudomembranes are transformed into firm adhesions leading to pachypleuritis. Open thoracotomy is the procedure of choice in patients with active bleeding and hemodynamic instability and was chosen for this patient.

## Conclusions

The diagnosis of SHP depends on recognizing the clinical patterns of sudden chest pain, dyspnea, shock, and clinical chest-localized signs. In particular, patients with SHP and spontaneous TP can have aggravated hemodynamic instability and hemorrhage. The presence of an air fluid line on CXR and the development of an hemorrhagic effusion and shock should alert the physician to this clinical entity. The successful treatment of a large SHP depends on early recognition, proactive intervention, and early consideration by a cardiothoracic surgeon. Once the diagnosis is confirmed, early thoracotomy should be considered. Notably, emergent surgery is required for SHP. Such an aggressive surgery not only leads to shorter hospitalization but also confers better long-term outcomes.

## Abbreviations

BNP: B-type natriuretic peptide
CXR: Chest X-ray
ER: Emergency room
PE: Physical examination
PSP: Primary spontaneous pneumothorax
PT: Prothrombin time
SHP: Spontaneous hemopneumothorax
SP: Spontaneous pneumothorax
TP: Tension pneumothorax
VATS: Video-assisted thoracoscopic surgery
